# Supplementary material for: Clinical Characteristics of Offspring Born to Parents with Type 2 Diabetes Diagnosed in Youth: Observations from TODAY
Source: Children (Basel). 2024 May 24;11(6):630. doi: 10.3390/children11060630 (PMC11201816; doi:10.3390/children11060630)
Supplement: Supplementary file 1 [file children-11-00630-s001.zip › children-3019329-supplementary.pdf]

Supplementary Material

Clinical Characteristics of Offspring Born to Parents with Type 2 Diabetes Diagnosed in Youth: Observations from TODAY

Jeanie B. Tryggestad, MD, Megan M. Kelsey, MD, Kimberly L. Drews, PhD*, Shirley Zhou, MS, Nancy Chang, PhD, Elia Escaname, MD, Samuel S. Gidding, MD, Elvira Isganaitis, MD, Siripoom McKay, MD, Rachana Shah, MD, Michelle Van Name, MD for the TODAY Study Group

*** Correspondence:** Corresponding Author: today@bsc.gwu.edu

Table S1. Comparison of TODAY baseline characteristics between those who completed the offspring questionnaire at the final study visit and those who did not by TODAY participant sex.

	**Females**			**Males**		
	**Excluded (N=153)**	**Included (N=299)**	**p-value**	**Excluded (N=89)**	**Included (N=158)**	**p-value**
Age (years)	14.0 (2.1)	13.6 (2.0)	**0.0258**	14.7 (1.8)	14.3 (1.9)	0.1428
Duration of T2D (months)	8.8 (6.0)	8.5 (6.1)	0.5966	7.1 (4.7)	8.2 (5.7)	0.1205
Race/Ethnicity (%)			**0.0482**			**0.0015**
White, non-Hispanic	26.8%	39.1%		25.8%	29.1%	
Black, non-Hispanic	45.1%	33.8%		41.6%	44.9%	
Hispanic	19.6%	18.7%		24.7%	21.5%	
Other	8.5%	8.4%		7.9%	4.4%	
Household income			**0.0014**			0.1029
<$25,000	30.1%	41.5%		32.6%	38.0%	
$25,000-$49,999	28.8%	33.1%		24.7%	28.5%	
>$50,000	30.1%	16.4%		32.6%	19.6%	
Unknown	11.1%	9.0%		10.1%	13.9%	
Highest household education			0.1023			0.0929
Less than high school	29.5%	24.6%		27.3%	27.1%	
High school degree or equivalent	19.9%	27.9%		18.2%	28.4%	
Some college	30.1%	33.3%		39.8%	25.8%	
College degree or higher	20.6%	14.1%		14.8%	18.7%	
HbA1c	6.1 (0.8)	6.0 (0.8)	0.8571	5.9 (0.7)	6.0 (0.8)	0.4930
BMI (kg/m^2)	34.6 (7.7)	34.5 (7.1)	0.8690	36.0 (7.7)	35.4 (8.4)	0.5914
BMI percentile	97.4 (3.6)	97.6 (3.3)	0.7400	98.2 (3.1)	97.9 (3.3)	0.4347

Table S2. Primary caregiver for the offspring by sex of participant and overall

	Mothers	Fathers	Overall
N (Number of offspring)	182	46	228
Caregiver-mother (N, %)	167 (91.76%)	40 (86.96%)	207 (90.79%)
Caregiver-father (N, %)	59 *(32.42%)*	28 (60.87%)	87 (38.16%)
Caregiver-grandmother-maternal (N, %)	34 (18.68%)	2 (4.35%)	36 (15.79%)
Caregiver-grandmother-paternal (N, %)	6 (3.30%)	1 (2.17%)	7 (3.07%)
Caregiver-grandfather-maternal (N, %)	19 (10.44%)	1 (2.17%)	20 (8.77%)
Caregiver-grandfather-paternal (N, %)	4 (2.20%)	0 (0.00%)	4 (1.75%)
Caregiver – Other (N, %)	34 (18.68%)	2 (4.35%)	36 (15.79%)
